# Expression and characteristics of manganese peroxidase from *Ganoderma lucidum* in *Pichia pastoris* and its application in the degradation of four dyes and phenol

**DOI:** 10.1186/s12896-017-0338-5

**Published:** 2017-02-23

**Authors:** Hui Xu, Meng-Yuan Guo, Yan-Hua Gao, Xiao-Hui Bai, Xuan-Wei Zhou

**Affiliations:** 1 Key Laboratory of Urban Agriculture (South) Ministry of Agriculture, and Engineering Research Center of Cell & Therapeutic Antibody, Ministry of Education, and School of Agriculture and Biology, Shanghai Jiao Tong University, Shanghai, 200240 People’s Republic of China; 2 State Key Laboratory of Microbial Metabolism, School of Life Sciences and Biotechnology, Shanghai Jiao Tong University, Shanghai, 200240 People’s Republic of China

**Keywords:** *Ganoderma lucidum*, Yeast expression system, Manganese peroxidase, Degradation, Phenolic compound

## Abstract

**Background:**

Manganese peroxidase (MnP) of white rot basidiomycetes, an extracellular heme enzyme, is part of a peroxidase superfamily that is capable of degrading the different phenolic compounds. *Ganoderma*, a white rot basidiomycete widely distributed worldwide, could secrete lignin-modifying enzymes (LME), including laccase (Lac), lignin peroxidases (LiP) and MnP.

**Results:**

After the selection of a *G. lucidum* strain from five *Ganoderma* strains, the 1092 bp full-length cDNA of the *MnP* gene, designated as *G. lucidum MnP* (*GluMnP1*), was cloned from the selected strain. We subsequently constructed an eukaryotic expression vector, pAO815:: *GlMnP*, and transferred it into *Pichia pastoris* SMD116. Recombinant GluMnP1 (rGluMnP1) was with a yield of 126 mg/L and a molecular weight of approximately 37.72 kDa and a specific enzyme activity of 524.61 U/L. The rGluMnP1 could be capable of the decolorization of four types of dyes and the degradation of phenol. Phenol and its principal degradation products including hydroquinone, pyrocatechol, resorcinol, benzoquinone, were detected successfully in the experiments.

**Conclusions:**

The rGluMnP1 could be effectively expressed in *Pichia pastoris* and with a higher oxidation activity. We infer that, in the initial stages of the reaction, the catechol-mediated cycle should be the principal route of enzymatic degradation of phenol and its oxidation products. This study highlights the potential industrial applications associated with the production of MnP by genetic engineering methods, and the application of industrial wastewater treatment.

**Electronic supplementary material:**

The online version of this article (doi:10.1186/s12896-017-0338-5) contains supplementary material, which is available to authorized users.

## Background

Manganese peroxidase (MnP) (E.C. 1.11.1.13. Mn^2+^: H_2_O_2_ oxidoreductases) belongs to the family of oxidoreductases, to be specifically those actions on peroxide as acceptor (peroxidases), is an extracellular hemeprotein which catalyze the H_2_O_2_-dependent oxidation of lignin-derivatives based polymers [1]. MnP is a specific enzyme that can oxidize Mn^2+^ to Mn^3+^, which diffuses from the enzyme surface and in turn oxidizes the phenolic substrate, including lignin model compounds and some organic pollutants [2]. In nature, MnP catalyzes plant lignin de-polymerization as component of ligninolytic enzymes complex. So it is one of the most common lignin degradation enzymes and has great application potential in the field of agriculture for degradation of some cellulose, hemicellulose and lignin, etc. To protect the environment, it was widely used in many industrial fields for degradation some recalcitrant organic pollutants such as polycyclic aromatic hydrocarbons, chlorophenols, industrial dyes and nitroaromatic compounds, which are very harmful to human health [3]. Recently, more and more attention has been paid to the value of bioremediation of this enzyme.

MnP was first discovered in *Phanerochaete chrysosporium* [4] and seems to be the most ubiquitous ligninolytic enzyme among white-rot fungi. At present, it has been purified and characterized from various white rot fungi [5–11]. Properties and application on MnPs isolated from different sources had been investigated widely. Much previous research has suggested that some azo dyes could be efficiently degraded by the purified MnPs, which were isolated from *P. chrysosporium*, *Lentinula edodes*, *Trametes versicolor*, *Dichomitus squalens*, *Stereum ostrea*, *Irpex lacteus* and etc. [3, 12–16]. However, many factors influenced the application of MnP, which include slow fungal growth rate, accumulation of extracellular polysaccharides, similar chromatographic properties of MnP and laccase, and etc. [17]. Therefore, searching for new MnP from widely distributed worldwide and fast fungal growth rate is essential for the application of MnP in industrial and agricultural productions, and environmental protection.

*Ganoderma*, a white rot basidiomycete widely distributed worldwide, can be cultivated on various substrates by different cultivation model, and could secrete lignin-modifying enzymes (LME), including laccase (Lac), lignin peroxidases (LiP) and MnP. Because of the rapid growth rate and extensive decolorization on solid media, Ganoderma is suitable for a wide range of applications in the field of environment and biotechnology; previous publications had reported that several species of *Ganoderma* can produce high amounts of MnP enzymes in solid or liquid cultures [2]. However, as we know, few studies focused their attention on the evaluation of the capability of purified and heterologous expression MnP tolerating different for dyes or other industrial pollutants. In the previous publications, most of them mainly focused on inducing secretion of MnP from different *Ganoderma*, and their potential uses in decolorization of textile effluents [18] and degrades β-carotene from *G. applanatum* under alkaline conditions [19].

In the present study, the possible difference of various *G. lucidum* strain for production of the MnPs was investigated using a qualitative plate assay method by using O-methoxyphenol as a color indicator. The fungal colony showing the largest zone of decolorization was selected for cloning the *MnP1* cDNA sequence, and then an expression vector, pAO815:: GluMnP1, was constructed and transferred into *P. pastoris* SMD1168H by electroporation-mediated transformation. The expression products were demonstrated by sodium dodecyl sulfate-polyacrylamide gel electrophoresis (SDS-PAGE) and western blotting. We also carried out a preliminary exploration on the ability of rGluMnP1 to biodegrade four dyes and phenol, and infer a probable degradation route of phenolic compounds, which should be taken into account in producing and designing a related industrial wastewater treatment process. This study provides a production strategy for MnP and will aid our understanding of the role of fungal MnP oxidation in biodegradation and bioremediation.

## Results

### Selection of the strain from various *G. species*

**Fig. 1:**
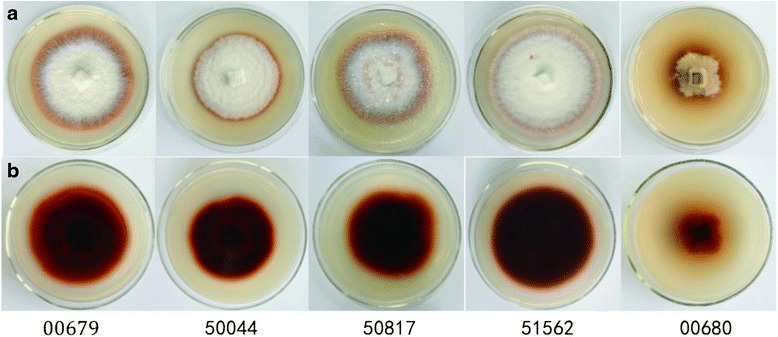
Decolorization of O-methoxyphenol with five *G. lucidum* strains. *G. lucidm* 00679, 50044, 50817, 51562 and 00680 was cultured on PDA medium for 7 d, and then was taken photographs. **a** displayed on the front of the petri dish, and (**b**) displayed the reverse side of the petri dish

**Fig. 2:**
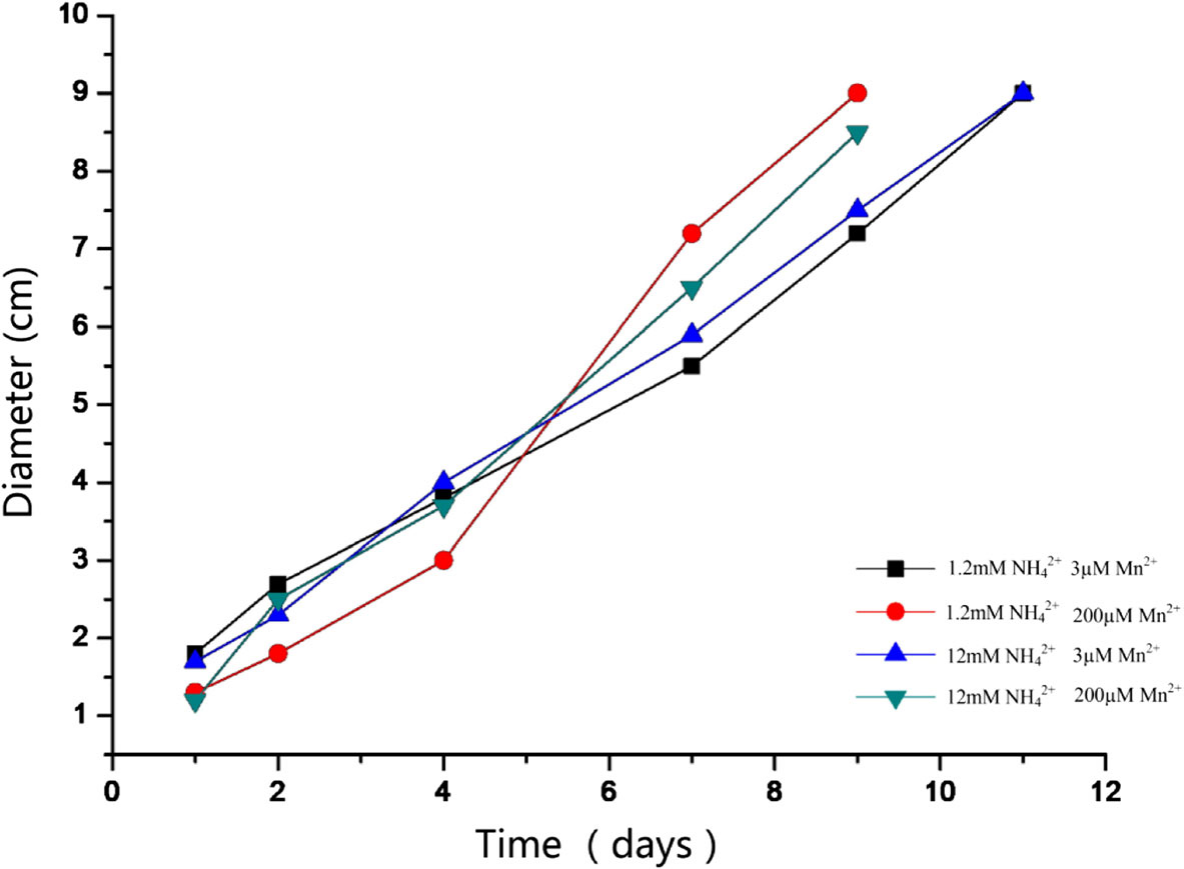
Diameters of colored red-brown circled with *G. lucidum* 00679 by N-limited and N-rich cultures at 3 μM and 200 μM Mn^2+^

The ability of producing lignin-degrading enzymes of five species of *G. lucidum* strains was measured by comparing the diameter of the colony and reddish brown circles. The results showed that the ratio of diameter of reddish brown circles and the diameter of fungal colony was the largest when *G. lucidum* 00679 was cultured for 7 days (Fig. 1). In order to better understand the lignin-degrading enzyme from *G. lucidum*, we tested the action of manganese peroxidase. At initial concentrations of 1.2 mM NH_4_^2+^ and 3 μM Mn^2+^, extracellular production of MnP and Lac began by day 4, with maximum levels of 1003 U/L Lac on day 14 and 57 U/L MnP on day 16. At a higher initial Mn^2+^ concentration of 200 μM, MnP and Lac production also began at day 4 with more MnP produced. The maximum level of Lac was less. No LiP was detected. The results showed that initial concentrations of 1.2 mM NH_4_^2+^ and 200 μM Mn^2+^, extracellular production of MnP of *G. lucidum 00679* with maximum levels, reached 670 (U/L) (Table 1). Despite significant differences in enzyme production, cultures at both Mn^2+^ concentrations rapidly colorful reaction in the PDA-O-methoxyphenol plate, with no difference in the ratio of diameter of reddish brown circles (Fig. 2). As a result, the fungal colony of *G. lucidum* 00679 for highest decolorization zone was chosen for the further study.

**Table 1:** MnP, Lac and Lip production by N-limited and N-rich batch cultures at 3 μM and 200 μM Mn^2+^

enzymes	1.2 mM NH_4_^2+^		12 mM NH_4_^2+^	
	3 μM Mn^2+^	200 μM Mn^2+^	3 μM Mn^2+^	200 μM Mn^2+^
Maximum MnP (U/L)	57	670	33	72
Maximum Lac (U/L)	1003	69	890	230
Maximum LiP (U/L)	undetected	undetected	undetected	undetected

### Decolorization of four dyes by the culture supernatants of *G. lucidum* strains

The results showed that *G. lucidum* 00679 could efficiently decolorize these four dyes. Drimaren Blue CL-BR, Drimaren Yellow X-8GN, Drimaren Red K-4Bl in the aqueous solutions (500 mg/L) were respectively decolorized up to 92.8, 90.2 and 70.1% by *G. lucidum* 00679 within 72 h. Disperse Navy Blue HGL in the aqueous solution (500 mg/L) could be decolorized up to 93.4% by *G. lucidum* 00679 within 12 h. MnP, Lac, and LiP activities were assayed in the supernatant medium before and after decolorization. Extracellular MnP activities were significantly induced by 278.1, 300.9, 259.3 and 191.3% respectively after decolorization of four dyes by *G. lucidum* 00679. Less lac and nor LiP was detected during the decolorization process. Induction in MnP activity during the decolorization process suggested that MnP was involved in the decolorization of these four dyes.

### Isolation and Sequence Characterization of the MnP gene

Based on total RNA isolated from the mycelia of *G. lucidum*, degenerate primers MnPF1 and MnPR1 [see Additional file 1] were used to specifically amplify a 461 bp core fragment using a method of the one-step real-time reverse transcriptase-PCR (RT-PCR) [see Additional file 2A]. A BLAST search showed that the PCR core fragment was homologous to *MnP* genes from other white rot fungi species (data not shown). The 5′ and 3′-ends fragments (222 and 870 bp, respectively) were amplified by 5′ RACE [see Additional file 2 B] and 3′ RACE [see Additional file 2C], based on the 461 bp core fragments. The core fragment, and the 3′- and 5′-ends fragments were assembled using Vector NTI Suite 10 and the deduced full-length *GluMnP1* cDNA sequence obtained was confirmed by sequencing. The full-length cDNA of *GluMnP1* was 1,341 bp [see Additional file 2D], comprising a 70 bp 5′-untranslated region, an ORF of 1095 bp and a 176 bp 3′-untranslated region.

Sequence analysis confirmed isolation of a full-length cDNA of *GlMnP1* encoding a protein of 364 amino acids, with a calculated molecular mass of 37.7 kDa and isoelectric point (pI) of 4.43. Amino acids of GlMnP1 involved in aromatic substrate oxidation on the distal side of the heme, Ca^2+^ side binding residues, heme pocket residues and Mn^2+^ binding site. These features suggested that *GlMnP1* encoded a probable manganese peroxidase. A database search with Blastx (https://blast.ncbi.nlm.nih.gov/Blast.cgi) showed that there was a relatively high similarity between *GluMnp1* and other *MnP*s from strains, such as *GluMnP*, *GapMnP*, *GfoMnP*, and *GauMnP*. A number of gaps and insertions were made in the sequences to optimize the alignment. The percentages of identity among *GluMnP*, *GauMnP*, and *GfoMnP* were 98, 88 and 87%, respectively, suggesting they were closely related to each other [see Additional file 3]. Amino acids of GluMnP1 involved in aromatic substrate oxidation were first analyzed and compared with various other plants and fungi by bioinformatics analysis [see Additional file 3]. Amino acids involved in aromatic substrate oxidation [see Additional file 3A] on the distal side of the heme, Ca^2+^ side binding residues [see Additional file 3C], heme pocket residues [see Additional file 3H] and Mn^2+^ binding site [see Additional file 3, M] were conserved in the MnP sequences from *G. lucidum* as well as in peroxidase sequences from various other plants and fungi. The deduced sequence contained eight cysteines [see Additional file 3C], which probably form four disulfide bonds in the mature protein.

### Heterologous expression of *GluMnP1* gene in *P. pastoris*

**Fig. 3:**
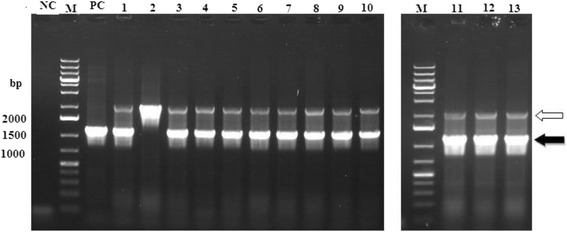
Electrophoresis of PCR amplification of *GluMnP1* from pAO815::*GluMnP1* Lane M: DNA marker DL 10000; lane NC: negative control; lane PC: positive control; Lane 1–13: selected transformants. The hollow arrow showed the DNA bands of AOX gene from yeast (about 2200 bp). The solid arrow showed the DNA bands of the *GluMnP1* gene plus a part of vector sequences (about 1300 bp)

**Fig. 4:**
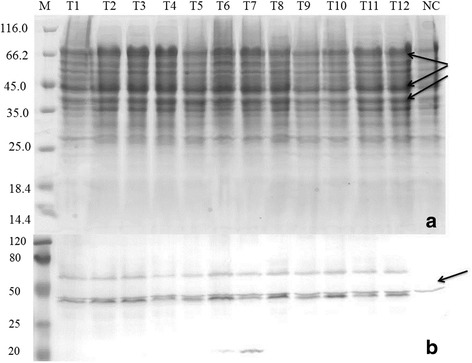
SDS-PAGE (**a**) and western blot analysis (**b**) of positive clones of recombinant *Ganoderma* MnP after 2 days of induction (**a**), lane M: protein marker; lanes 1–13: recombinant plasmid pAO815::MnP; Lane NC: negative control. Arrows showed the expressed bands. **b**, lane M: protein marker; lanes 1–13: re-pAO815-MnP; lane NC: negative control; lane PC: positive control. An arrow indicated the target band

The presence of *GluMnP1* in the transformants was confirmed by PCR (Fig. 3). SDS-PAGE analysis after Coomassie Brilliant Blue R-250 staining indicated that rGluMnP1 could be efficiently expressed in *P. pastoris* cells (Fig. 4a). The theoretical mass of the target rGluMnP1 protein was 38 KDa, and the mass of the rGluMnP1 protein after glycosylation modification was higher than the theoretical value.

The size of expressed protein was analyzed by western blotting of samples from a three-day fermentation of the yeast. The results of western blot analysis showed that the target protein of GluMnP1 from *G. lucidum* was heterologously expressed in *P. pastoris* (Fig. 4b).

### Analysis of enzyme yield and activity

The content of total soluble protein was determined approximately 1258 mg/L using the Bradford Protein Assay Kit. The density of protein bands was detected using the software Bandscan 5.0 (Glyko, Novato, USA) and the rGluMnP1 protein was estimated to account for about 10% of total soluble protein. Therefore, the yield of rGluMnP1 produced by the yeast transformants reached roughly 126 mg/L.

Methanol was used to induce expression in the *P. pastoris* transformants in BMMY medium. After fragmenting of *P. pastoris* and centrifugation, the highest rGluMnP1 activity in the culture supernatant of total protein extracted from *P. pastoris* transformants reached about 524.61 U/L after 48 h of incubation.

### Decolorization of four dyes using the rGluMnP1

**Fig. 5:**
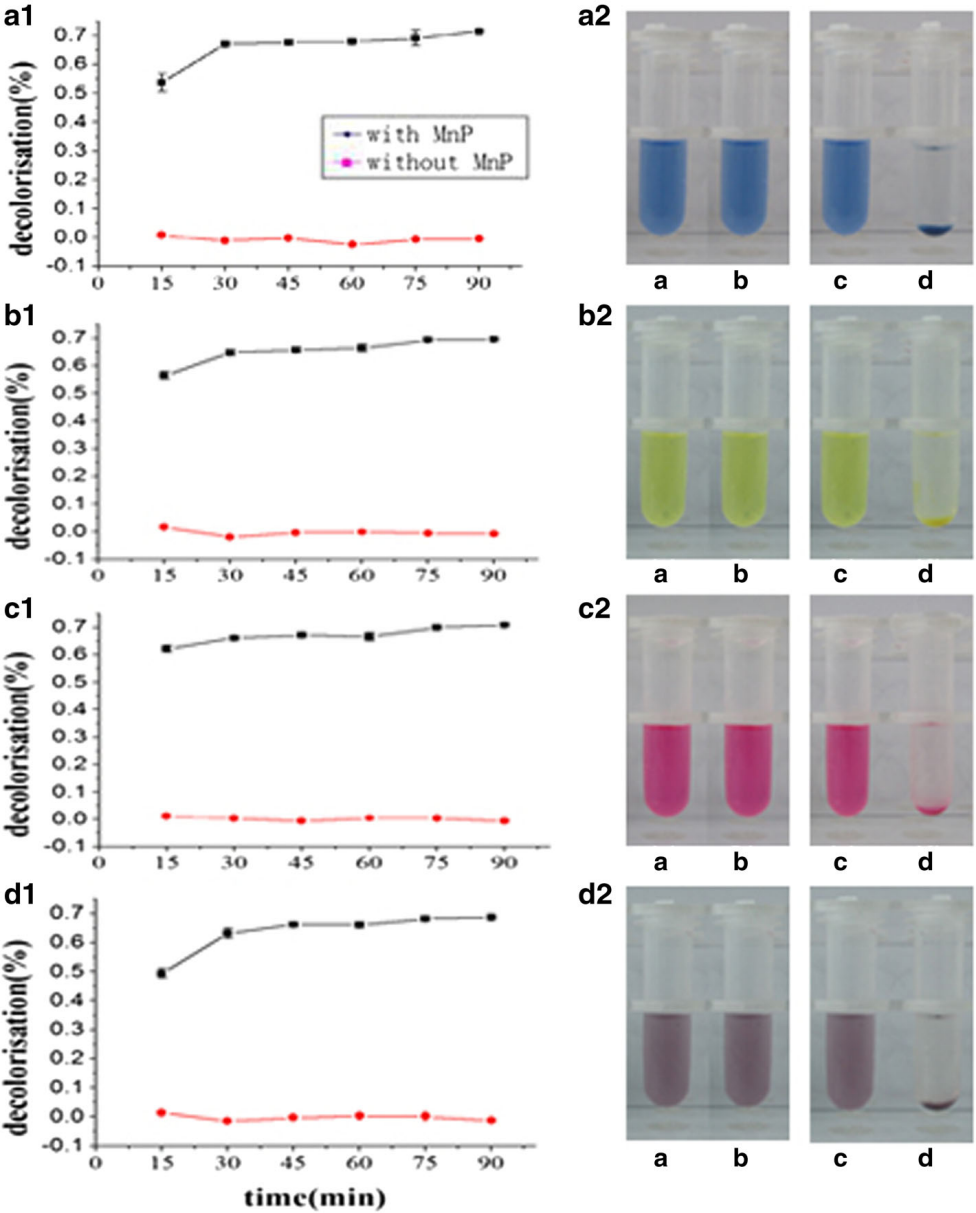
Time course and visual effect of decolorization of four dyes by the crude enzymatic solution. A1, B1, C1 and D1 show the in vitro decolorization rate of four dyes over time by consumption of crude rGluMnP1, which included Drimaren Blue CL-BR (A1), Drimaren Yellow X-8GN (B1), Drimaren Red K-4Bl (C1) and Disperse Navy Blue HGL (D1). The black line in each image shows the decolorization rate after treatment by rGluMnP1; the red line shows the results for the negative control. A2, B2, C2 and D2 showed the visual decolorization effect of four dyes by control (untransformed yeast) and crude rGluMnP1 solutions (transformed yeast). (a) and (b) show the visual decolorization effects before/after treated by the untransformed yeast, (c) and (d) show the visual decolorization effects before/after treated by the yeast transformants. Yeasts were broken to prepare the crude enzyme solutions. The reactions were carried out in a 2 mL EP tube

The decolorization experiments were performed with crude protein extracts in 50 mM sodium malonate (pH 4.5) and 500 mg/L of dyes in a final volume of 1 mL at 25 °C. The results showed that the maximum decolorization rates of the four dyes all reached 70% (Fig. 5), indicating that rGluMnP1 had a higher decolorizing ability. Reaction containing MnSO_4_ and H_2_O_2_ could only decolorize four dyes by about 49% in 15 min. However, rGluMnP1 could decolorize Drimaren Red K-4Bl by more than 62% after 15 min. The decolorization rates of the four dyes increased quickly at the start of the reaction, but increased more slowly after 30 min.

### HPLC-based analysis of the degradation rate of phenol and the principal degradation products

**Fig. 6:**
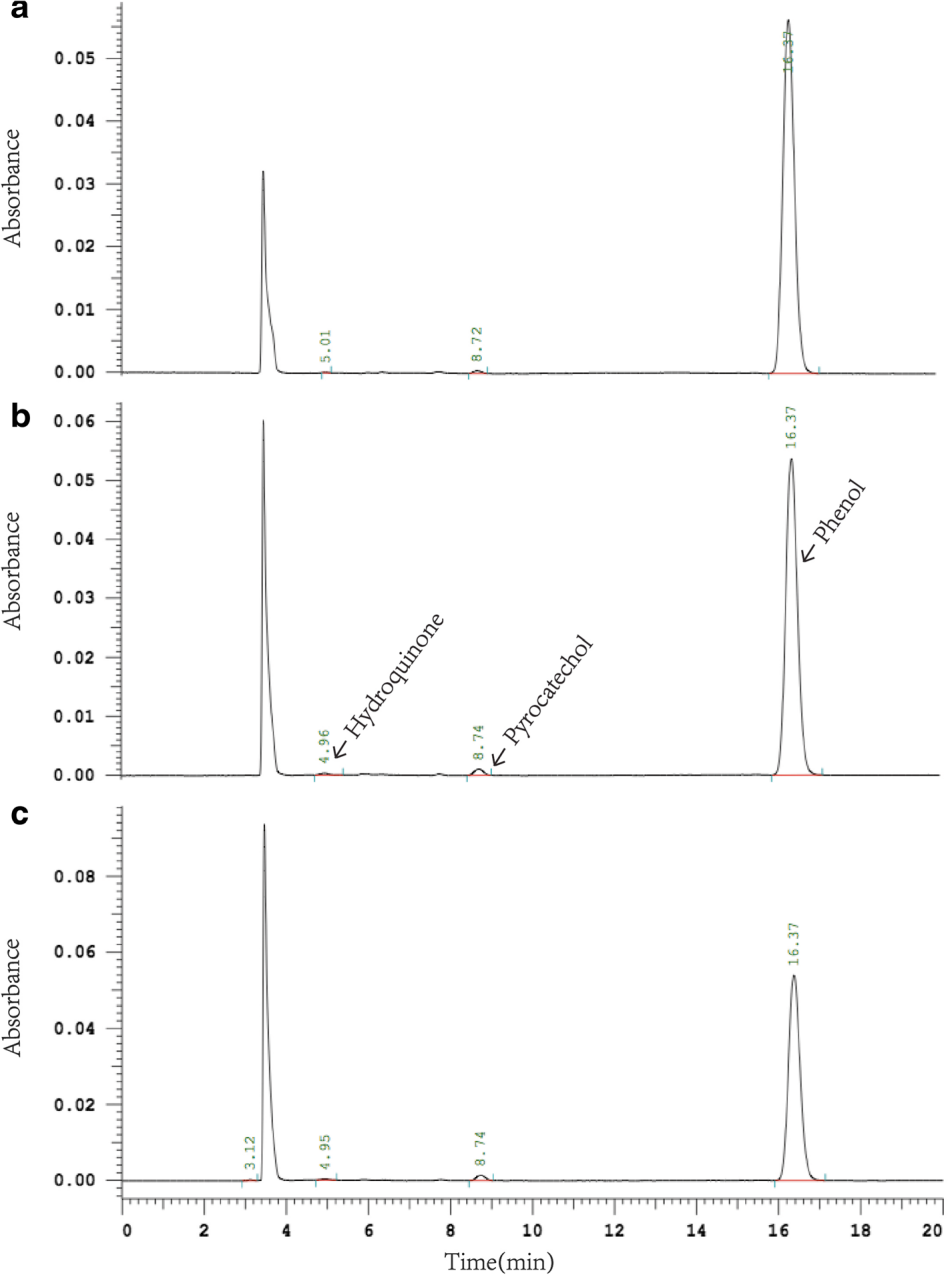
HPLC chromatography of phenol and the main degradation products. HPLC analysis of the phenol (retention time = 16.37 ± 0.1 min) and its main degradation products of hydroquinone (retention time = 4.96 ± 0.1 min), pyrocatechol (retention time = 8.74 ± 0.1 min). The aqueous solutions of phenol were treated by 5% (Fig. 6a, upper spectrum), 10% (Fig. 6b, middle spectrum) and 15% (Fig. 6c, lower spectrum) rGlMnP1 enzyme solutions, respectively

Based on the establishment of the standard curves of phenol and the principal degradation products (data not shown), the regression equations of calibration curves and their coefficients were calculated as follows: for phenol, Y = 6046.4X + 21365 (R^2^ = 0.9998); for hydroquinone, Y = 11297X − 3710.4 (R^2^ = 0.9999); for pyrocatechol, Y = 13955X − 20052 (R^2^ = 0.9999). The high performance liquid chromatography (HPLC) analysis results showed that rGluMnP1 solutions can degrade phenol in aqueous solution effectively and the degradation products contained hydroquinone and pyrocatechol at least (Fig. 6). Furtherly, the contents of phenol in the samples dealt with by the crude enzymes solutions at the concentration of 5, 10 and 15% were about 88.914 ± 0.958, 84.642 ± 1.478 and 84.258 ± 1.613 μg/mL, respectively. The degradation rates of phenol in aqueous solution treated by 5, 10 and 15% crude enzymes solutions were 7.262 ± 0.999%, 8.079 ± 1.605% and 4.873 ± 1.821%, respectively. The results suggested that optimum concentration of crude enzymes solutions was about 10% under the present conditions.

The HPLC analysis results of the likely oxidation products of phenol showed that among the four possible degradation products including hydroquinone, pyrocatechol, resorcinol and benzoquinone, only two chemical compounds, hydroquinone and pyrocatechol were determined in the treated sample, the others were not determined by HPLC (Fig. 6). The contents of hydroquinone and pyrocatechol in the sample treated by crude enzymes solutions at the concentration of 5, 10 and 15% were found to be 0.359 ± 0.053, 0.517 ± 0.028, 0.503 ± 0.124 μg/mL and 1.640 ± 0.047, 2.014 ± 0.123, 2.180 ± 0.137 μg/mL, respectively.

## Discussion

For screening process on the ability of producing LMEs from different *G. lucidum* strains by comparing the diameter of the colony and reddish brown circles, based on previous theory, the smaller the ratio is, the stronger the strains’ ability of producing lignin-degrading enzymes is [20]. As a result, *G. lucidum* 00679 selected for the further study should be correct. Previous studies had demonstrated that Mn peroxidase production was controlled by the concentration of Mn^2+^ [21]. At higher Mn^2+^ concentrations, production of MnP increased and that of laccase decreased, but the rate or number of decolozations was unaffected [22]. In addition, the nitrogen source and its concentration were found to influence MnP production [23]. In order to obtain more information about the MnP from *G. lucidum*, Mn and nitrogen concentration were routine used to test the effects of MnP production and activity in the present study.

White rot fungi have been widely studied over the last 30 years because they could release LMEs and had a high capacity for biodegradation of environmental pollutants [24]. Most white-rot basidiomycetes are capable of degrading or oxidizing a range of aromatic organic compounds with the aid of certain enzymes, such as LiP, MnP and other versatile peroxidases [25]. To date, more than thirty enzymes have been isolated and investigated from at least a dozen *Ganoderma* species*.* MnP, as one of major fungal oxidative enzymes, plays a key role in enzymatic degradation of phenolic compounds in vitro. There are many reports concerning the decolorization of wastewater from dyeing factories [2, 26]. However, high-level expression of MnP must be taken into account before it can be used commercially. MnP expression level in some isolates is too low for industrial application. Heterologous expression in *P. pastoris* could meet these requirements, because it enhanced the expression levels by 10-, 100-, or even 1000-fold compared with the natural host [27].

*G. lucidum* contained 7 peroxidases genes in its genome, was the third largest number of peroxidases, which may suggest its strong ligninolytic ability [28]. In order to better use *Ganoderma* MnP for the degradation of different phenolic compounds, the MnP gene of and its full-length cDNA were successfully cloned and characterized from *G. lucidum* 00679. In the process of yeast expression, the complexity of the yeast intracellular proteins meant that the target protein would migrate with other proteins, possibly resulting in no significant band being observed on SDS-PAGE; however, as the recombinant expressed protein is expressed at a higher level than similar sized endogenous proteins, an individual protein band was observed that was not present in the negative control, indicating expression of the target protein (Fig. 4). For the yield or enzymatic activity of protein expression, compared with the native host, recombinant fungi and yeast strains could produce from 5 to 100 mg/L rMnP [29]. Comparing with the results of previous studies [30], in the present study, the highest rGluMnP1 activity in the culture supernatant of total protein extracted from *P. pastoris* transformants reached about 524.61 U/L at 25 °C and pH 4.5.

Peroxidases are hemoproteins that catalyze reactions in the presence of hydrogen peroxide. MnPs have a reaction mechanism that starts with enzyme oxidation by H_2_O_2_ to an oxidized state during the catalytic cycle [31]. The degradation mechanism of LMEs has been studied extensively using different white rot fungi [32, 33]. Zhang et al. (1999) demonstrated that MnP plays an important role in the decolorization of cotton bleaching effluent by an unidentified white-rot fungus, while there was no obvious role for LiP in this decolorization [34]. In other words, the relationship between these fungal oxidative enzymes in the decolorization process is not clear. However, it is likely that the main enzymes that decolorize different dyes are not the same [35, 36]. In 2005, Champagne and Ramsay approved that the combination of the MnP and Lac had an additive effect, and that MnP was the principal active enzyme in the reaction process [14]. Although H_2_O_2_ or ^−^OH may oxidize a dye without an enzyme, the decolorization of dyes mainly relies on Mn^3+^ acting on the organic acid compounds, which were also demonstrated in this study. In addition, based on the opinions of previous literature [37], a crude enzymatic solution was used to decolorize the four dyes in the present study, which could narrow the high costs associated with enzyme purification.

**Fig. 7:**
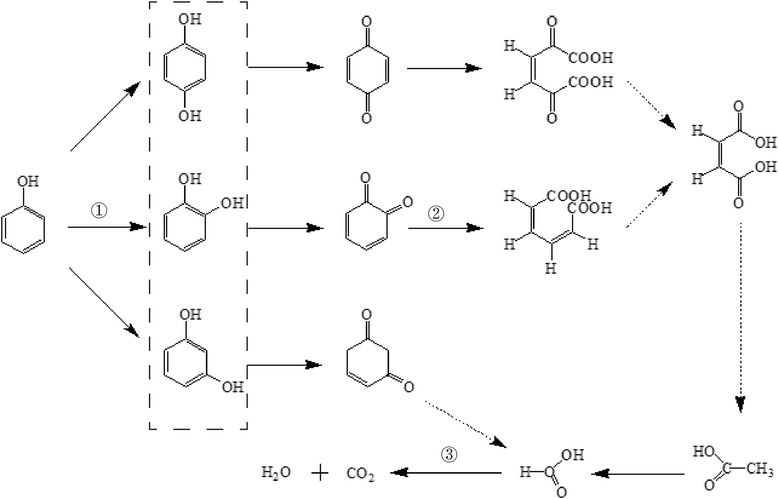
Pictorial scheme of the enzymatic degradation route of phenol. ① Hydroxylation of benzene formed the dihydroxybenzene and quinones. ② Dihydroxybenzene and quinones dehydrogenated and opened loop to form carboxylic acids. ③ Carboxylic acids mineralized to carbon dioxide and water. Dashed frame represented the compounds that were detected in this experiment

The above analysis had proved that the hydroxyl radical played a major role in phenol degradation. Resorcinol and benzoquinone were not found in the probable oxidation products due to the in perfect reaction system or in appropriate reaction conditions. We infer that the results are relevant to the phenol structure. The electronic arc in phenolic hydroxyl oxygen atom can have p-π conjugation with π electronic in the phenyl ring which made phenyl ring had more negative charge on the ortho- and/or para- in phenolic hydroxyl. So it is not difficult to produce hydroquinone and pyrocatechol by hydroxyl radical attack. The carboxylation of the phenyl ring is the first step of phenolic compounds degradation. Subsequently, the phenyl ring was open for the formation of carboxyl aromatic ring, and finally was completely mineralized to carbon dioxide and water (Fig. 7) [38]. Phenol intermediate products and its degradation route were needed for further investigation and analysis.

## Conclusions

In this study, we found *Ganoderma* strains with a capacity of the decolorisation of four types of dyes, cloned a *MnP* gene from *G. lucidum* 00679 and expressed this gene in the methylotrophic yeast *P. pastoris* that produced an intracellular rGluMnP1 with a stable and active form. The higher oxidation capacity of the recombinant proteins was established by using the enzymes for the decolorization of four dyes and the degradation of phenol, in which phenol and the main degradation products were especially confirmed by HPLC. From this result, we inferred that the degradation of phenolic compounds may relate to the phenol structure. In the initial stages of the reaction, this catechol-mediated cycle should be the principal route of enzymatic degradation of phenol and its oxidation products. In summary, the rGluMnP1 showed a great potential for the enzymatic degradation of industrial dyes and phenolic compounds.

## Methods

### Strains, plasmids and media

*G. lucidum* strain 51562, 50044, 00679, 50817, 00680 were purchased from the Agricultural Culture Collection of China (ACCC) (Beijing, China). *Escherichia coli* DH5α were preserved by the Plant Biotechnology Research Center, School of Agriculture and Biology, Shanghai Jiao Tong University (Shanghai, China). *P. pastoris* strain SMD1168H and the pAO815 yeast expression vectors were purchased from Invitrogen (San Diego, CA, USA).

Potato dextrose agar (PDA) medium was used to culture *Ganoderma* species. Four different kinds of media, yeast extract peptone dextrose (YPD) medium, minimal dextrose (MD) medium, buffered minimal glycerol-complex (BMGY) medium and buffered methanol-complex (BMMY) medium, were used to culture *P. Pastoris* [29, 37].

### Preparation and selection of fungal strains

The culture of *G. lucidum* mycelia was based on those described in the previous literature [39]. To select the suitable strain, stock cultures of different *G. lucidum* strains (51562, 50044, 00679, 50817, and 00680) were maintained in slant tubes at 4 °C on improved PDA medium (potato 200 g/L, dextrose 20 g/L, MgSO_4_ · 7H_2_O g/L, KH_2_PO_4_ 2.5 mg/L, vitamin B_1_ 10 mg/L, agar 20 g/L). Stock cultures were transferred onto agar plates containing the improved PDA medium and allowed to incubate for 5 d at 28 °C. Subsequently, agar blocks of the same size with the activated mycelia were cut from the edges of the growing colonies on the agar plates covered by the mycelia. Cut cultures were then transferred onto the Petri dishes containing the improved PDA medium containing 1 g/L O-methoxyphenol and allowed to incubate at 28 °C for 7 days. The diameters of the respective colonies and the decolorized zones were observed on the 13^th^ day [21].

To screen the Mn influence on the MnP production, the mycelia suspension (0.5 mL) was added into 500 mL Erlenmeyer flasks containing 200 mL of liquid PDA medium. Two media, N-rich (12 mM ammonium tartrate) and N-limited (1.2 mM ammonium tartrate) PDA media with 3 μM and 200 μM Mn^2+^, were established by adding appropriate amounts of MnSO_4_ · H_2_O. Furtherly, 30 mL portions of inoculum were inoculated into 200 mL of medium in 500 mL Erlenmeyer flasks, then the cultures were incubated at 28 °C with 200 rpm. 1 g/L O-methoxyphenol was added to flasks on day 0. All batch experiments in the current study were done in duplicate; results were reported as the average of analyses of triplicate sample. For decolorization of four dyes by the culture supernatants, the culture supernatants prepared from *G. lucidum* 00679 were used to decolorize four dyes. The assays were performed at 28 °C. The reaction mixture in a total volume of 1 mL contained (final concentration): dyes (Drimaren Blue CL-BR, Drimaren Yellow X-8GN, Drimaren Red K-4Bl and Disperse Navy Blue HGL: 500 mg/L) and 100 μL culture supernatant.

### Cloning and Expression of MnP

Total RNA was extracted from 1.0 g of freshly harvested *G. lucidum* mycelia using a TIANGEN RNA prep pure plant kit (Tiangen Biotech Co. Ltd., Beijing, China). Total RNA was reverse-transcribed into cDNA using the PrimeScript®RT Master Mix Perfect Real Time, according to the manufacturer’s instructions (TaKaRa Biotechnology Co., Ltd., Dalian, China). The core fragment of *MnP* gene was cloned according to standard protocols of the one step R-T PCR kit (AMV) (TaKaRa, Dalian, China) using forward primer MnPF1 and reverse primer MnPR1 that were designed according to the conserved regions of the *MnP* gene of *Ganoderma* sp*.*, such as *G. lucidum* (ACA48488), *G. formosanum* (ABB77243), *G. applanatum* (BAA88392) and *G. australe* (ABB77244) deposited in GenBank. Following this step, 5′- and 3′-ends fragments were conducted using SMART technology (SMART^TM^ RACE cDNA Amplification Kit) to produce a full-length MnP sequence. Two gene-specific primers 3GlMnPF1 and 3GlMnPF2 were used only for 3′-ends fragments of *GlMnP*, and two gene-specific primers 5GlMnPR1 and 5GlMnPR2 for 5′-ends fragments. All amplified PCR products were purified, sub-cloned with the pMD 18-T vector system (TaKaRa, Dalian, China) and then sequenced. By aligning and assembling the products of the 3′-ends fragments, 5′-ends of the fragments and the core fragment, the full-length *MnP* sequence of *G. lucidum* was deduced and subsequently amplified using primers GlMnPFullF1 and GlMnPFullR1. All the primers [see Additional file 1] employed in PCR amplification were synthesized by the Shanghai Sangon Biotech Co. Ltd. (Shanghai, China).

After digestion with *Hin*dIII and *Eco*RI, digested products encoding *GluMnP1* gene were sub-cloned directly into vector pAO815 that was predigested with the same restriction enzymes. The ligation products were transformed into *E. coli* strain DH5α and transformants were confirmed by PCR. The resulting recombinant plasmid, designated as pAO815::*GluMnP1*, was then sequencing.

Competent *P. pastoris* cells were prepared using the Invitrogen EasySelecte™ Pichia Expression Kit (Invitrogen), according to manufacturer’s instructions. 80 μL of *P. pastoris* cells were transformed with 20 μL of pAO815::*GluMnP1*, previously linearized with *Pol*, as described in the instruction of Multi-Copy Pichia Expression Kit (Invitrogen, Carlsbad, USA). Transformed clones were selected on MD (Mininal Dextrose) medium with ampicillin at 0.5, 1.0 and 2.0 mg/mL. Genomic DNA was extracted from *P. pastoris* using the Yeast DNA Isolation Kit (Sangon, Shanghai, China) according to the manufacturer’s instructions. The transformants were further confirmed by PCR amplification of the *GluMnP1* gene, using the same primers used to clone it and with the AOX forward and reverse primers supplied with the kit. *P. pastoris* SMD1168 was used as a positive control and sterile water was used as a control.

### Induction time screening and western blot analysis

In order to find out the optimum time points and analyze the time-course of expression, 1 mL cultures were induced to express rGluMnP1 by the addition heme to 1 mM and MnSO_4_ to 0.5 mM and supplemented methanol to 1% each 24 h until the transformants OD_600_ reached to 5. Samples of the cultures were taken out at 24, 48 and 72 h and disrupted using a high-pressure homogenizer (APV-2000, Germany). The culture supernatant was then harvested by centrifugation. The expression product was extracted using a commercial Kit (BSP013; Sangon), and resuspended in 100 μL of 2 × SDS-PAGE sample buffer and boiled for 5 min at 95 °C. The samples were prepared according to the previous description, and analyzed by 15% SDS-PAGE and stained with Coomassie Brilliant Blue R-250 [40].

### Estimation of total protein and determination of enzyme activities

After being induced and cultured for 48 h, the recombinant *P. pastoris* transformants were harvested by centrifugation (4000 × g for 5 min at 4 °C). Cells were then resuspended in PBS buffer and disrupted by a high pressure homogenizer. After centrifugation, the supernatant was collected and determination of the protein content was based on a modified Bradford method [41]. To quantitatively analyze the relative concentrations of the expressed rGlMnP1 in cell supernatants was from the densitometry of the bands using the software BandScan 5.0 (Glyko, Novato, USA).

MnP activity was estimated by monitoring the oxidation of Mn^2+^ to Mn^3+^ at 270 nm using a UV/Visible spectrophotometer (DU 800, Beckman, USA), according to the Wariishi’s method [42]. The enzyme reaction system contained 0.5 mL sodium malonate (100 mM, pH 4.5), 0.1 mL of MnSO_4_ (10 mM) and 0–50 μL crude enzyme. The mixtures were incubated in 1.5 mL centrifuge tubes at 28 °C for 30 min. The reaction was started by adding 10 μL H_2_O_2_ (10 mM). The absorbance was immediately measured at 270 nm after the reactions were initiated using the addition of H_2_O_2_ to a concentration of 10 μM at room temperature. Both Lac and LiP activities were monitored as previously described [43].

### Decolorization of dyes by the rGluMnP1

Four dyes, Drimaren Blue CL-BR, Drimaren Yellow X-8GN, Drimaren Red K-4Bl and Disperse Navy Blue HGL, were used for enzymatic decoloration treatment. Decolorization reactions were carried out at room temperature. The disappearance of the dyes was determined spectrophotometrically (Evolution 300UV-VIS spectrophotometer, Thermo Scientific) by measuring the absorbance at the wavelength of the maximum absorbance for 150 mg/L of each dye [13]. The maximum absorbance of the four dyes are 590 nm, 425 nm, 540 nm, 570 nm respectively. Typically, 0.4 mL dye (500 mg/L) and 0.1 mL crude enzyme were added to 0.39 mL sodium malonate buffer (100 mM, pH 4.5) containing 0.1 mL of MnSO_4_ (1 mM). The buffer was used as a negative control. Reactions were initiated by adding 10 μL of H_2_O_2_ (100 μM) to the reaction mixture. Decolorization was followed spectrophotometrically by a microplate reader (Power wave XS, Bio-tek) using the maximum absorbance curves recorded under these conditions. The decolorizing change of each dye was calculated every 15 min for 90 min. The decolorization percentage was calculated using a method described in the previous literature [44]. Decolorization percentage A% = (A_0_-A)/A_0_ × 100% (A_0_-initial absorbance; A-final absorbance). All of the experiments were performed using three replicates and were repeated at least twice. The data presented in the text correspond to the mean values.

### HPLC-based analysis of phenol degradation rate and its oxidation products

The analysis of phenol and its oxidation products were carried out with a HPLC (Waters Corporation, Milford, MA, USA). Phenol, hydroquinone, pyrocatechol, resorcinol, benzoquinone and water were all the HPLC-grade and purchased from Sinopharm Chemical Reagent Co., Ltd. (Shanghai, China). The standard phenolic compounds including phenol, hydroquinone, pyrocatechol, resorcinol, benzoquinone solvent were consecutively injected five times to draw calibration curves. The injection volume was 2, 4, 8, 16 and 22 mL, respectively.

The oxidation reaction was carried out as previously described using 50 mL EP tubes [45]. A solution of 500 mg/L of simulation phenol-containing wastewater was made up with dissolving 1.00 g phenol at the constant volume of 2000 mL distilled water. The solution was sterilized at 121 °C for 20 min and then cooled to 30 °C. 5 mL phenol solutions were added into 50 mL EP tubes and add 5, 10 and 15% rGluMnP1 crude enzymes. Reactions are completely at 210 rpm/min and 28 °C for more than 24 h. The determination condition of the samples was set as follows: the column was YMC-pack ODS-A 250 × 4.60 mm S-5.0 μm; the mobile phase adopted in the analysis consists of methanol and water was in the ratio 30:70 (*v/v*). The separation was conducted in isocratic elution at a flow rate of 0.8 ml/min. The detection wavelength of photo-diode array was set at 280 nm; the column temperature was 30 °C. The injection volume was 20 μL and a data acquisition time of 20 minutes was used [46]. The degradation rate of phenol was calculated according to the following formula: Degradation rate (100%) = (C_0_-C)/C_0_ × 100%. In the formula, C_0_ was the concentration of phenol in the control group; C was the concentration of residual phenol in the treated phenol aqueous solution.

All experiments were performed at least twice using three replicates. The data presented in the text corresponded to the mean values.

## Additional files

Additional file 1: Primers used in this study. (DOC 30 kb)
Additional file 2: Electrophoresis photos of *GluMnP1* gene cloning from *G. lucidum* 00679. (DOC 79 kb)
Additional file 3: Multiple alignment of the amino acid sequences of GluMnP1 from *G. lucidum* 00679. (DOC 45 kb)

## Abbreviations

BMGY: Buffered minimal glycerol-complex
BMMY: Buffered methanol-complex
GluMnP1: MnP1 from *G. lucidum*
Lac: Laccase
LiP: Lignin peroxidase
LMEs: Lignin-modifying enzymes
MD: Minimal dextrose
MnP: Manganese peroxidase
PDA: Potato dextrose agar
rGluMnP1: Recombinant GluMnP1
SDS-PAGE: Sodium dodecyl sulfate-polyacrylamide gel electrophoresis
YPD: Yeast extract peptone dextrose
