# Supplementary material for: Expression and characteristics of manganese peroxidase from Ganoderma lucidum in Pichia pastoris and its application in the degradation of four dyes and phenol
Source: BMC Biotechnol. 2017 Feb 23;17:19. doi: 10.1186/s12896-017-0338-5 (PMC5324234; doi:10.1186/s12896-017-0338-5)
Supplement: Additional file 1: — Primers used in this study. (DOC 30 kb) [file 12896_2017_338_MOESM1_ESM.doc]

**Additional file 1**

Primers used in this study

| Primers | Sequence (5′----3′) |
| --- | --- |
| GlMnPF1 | ATGTTCTCA/CAAAGTCTTCCTCTCCCTCGTCGTCC |
| GlMnPR1 | AAGAACGAGA GCTGGGGG/ACCC/A/GCCGTTGCAGTT |
| 5GlMnPR1 | AATTGACCTGCCGCAGTCAGAGCAGGAG |
| 5GlMnPR2 | CTGGATGTCGTCGAGGACGTCGAACC |
| 3GlMnPF1 | cgacgacatccaggagaacctgttcc |
| 3GlMnPF2 | cgacgacatccaggagaacctgttcc |
| GlMnPFullF1 | tcgagcaccacctcgactctcggaga |
| GlMnPFullR1 | CACGACTACATAACAGATCAATAA |
