# Supplementary material for: Expression and characteristics of manganese peroxidase from Ganoderma lucidum in Pichia pastoris and its application in the degradation of four dyes and phenol
Source: BMC Biotechnol. 2017 Feb 23;17:19. doi: 10.1186/s12896-017-0338-5 (PMC5324234; doi:10.1186/s12896-017-0338-5)

**Additional file 2**

Electrophoresis photos of *GluMnP1* gene cloning from *G. lucidum* 00679.

Note: A-Electrophoresis photos of amplified middle fragment of *GluMnP1* gene, lane 1 shows amplification bands; B-Electrophoresis photos of amplified 5’-end fragment of *GluMnP1* gene, lane 1 shows the primary PCR of 5’-end fragment, 2 shows the nested PCR of 5’-end fragment; C-Electrophoresis photos of amplified 3’-end fragment of *GlMnP* gene, lane 1 shows the primary PCR of 3’-end fragment, 2 shows the nested PCR of 3’-end fragment; D-Amplified full length gene. Lane 1 shows the full length fragments of *GluMnP1* gene.


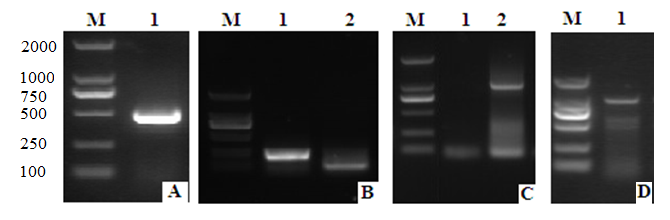

Supplement: Additional file 2: — Electrophoresis photos of GluMnP1 gene cloning from G. lucidum 00679. (DOC 79 kb) [file 12896_2017_338_MOESM2_ESM.doc]
