# Supplementary material for: Expression and characteristics of manganese peroxidase from Ganoderma lucidum in Pichia pastoris and its application in the degradation of four dyes and phenol
Source: BMC Biotechnol. 2017 Feb 23;17:19. doi: 10.1186/s12896-017-0338-5 (PMC5324234; doi:10.1186/s12896-017-0338-5)
Supplement: Additional file 3: — Multiple alignment of the amino acid sequences of GluMnP1 from G. lucidum 00679. (DOC 45 kb) [file 12896_2017_338_MOESM3_ESM.doc]

**Additional file 3**

Multiple alignment of the amino acid sequences of GluMnP1 from *G. lucidum* 00679*.* Peroxidase residues important for structure or catalytic activity are shown on a gray background and explained in the bottom line: A, aromatic substrate oxidation; B, disulfide bridge; C, Ca^2+^ binding residues; H, heme pocket residues; M, Mn^2+^ binding site.

Note: GluMnP1-MnP1 from *G. lucidum* 00679; GluMnP- MnP from *G. lucidum* (GenBank: ACA48488); GfoMnP- MnP from G. *formosanum* (ABB77243); GapMnP- MnP from *G. applanatum* (GenBank: BAA88392); GauMnP- MnP gene from *G. austral* (GenBank: ABB77244). Yellow: the cloned MnP gene possessing the same amino acid sequences with the gene from other *G. species*. Blue: the cloned MnP gene possessing the same part of amino acid sequences with the gene from other *G. species*. Colourless: the cloned MnP gene possessing the different amino acid sequences with the gene from other *G. species*.

*** ** * ***

GluMnP1 (1) MFSKVFLSLVVLAASVTAAVPSVGRRATCSNGKTTANDACCVWFDVLDDIQENLFHGGECGEDAH

GluMnP (1) MFSKVFLSLVVLAASVTAAVPSVSRRATCSNGKTTANEACCVWFDVLDDIQENLFHGGECGEDAH

GfoMnP (1) MFSKVFLSLVVLAASAAAAVPSVSRRATCSNGKTTANDACCVWFDVLDDIQENLFHGGQCGEDAH

GapMnP (1) MFSKVFLSLVVLASSVAAAVPTVGRRATCANGKTTANDACCVWFDVLDDIQENLFHGGQCGEDAH

GauMnP (1) MFSKVFLSLVVLASSVAAAVPSVGRRATCANGKTTANDACCVWFDVLDDIQENLFHGGQCGEDAH

**B BB B M**

*** * *** * * * * ***

GluMnP1 (66) ESLRLTFHDALGFSPALTAAGQFGGGGADGSIMAHSDVELTYPGNNGLDEIIEASRFFAIKHNVS

GluMnP (66) ESLRLTFHDALGFSPALTAAGKFGGGGADGSIMAHSDVELTYPGNNGLDEIIEASRPFAIKHNVS

GfoMnP (66) ESLRLTFHDAIGFSPALTAAGQFGGGGADGSIIAHSDVEMTYPANDGLDEIIEASRPFAIKHNVS

GapMnP (66) ESLRLTFHDAIAFSPALTAAGQFGGGGADGSIIAHSDVELTYPVNDGLDEIVEASRPFAIKHNVS

GauMnP (66) ESLRLTFHDAIAFSPALTAAGQFGGGGADGSIIAHSDVELTYPVNDGLDEIVEASRPFAIKHNVS

**M H HHC C C C H H**

*** ***

GluMnP1(131) FGDGIQFAGAVGAANCNGGPQLSFFAGRSNDSQPSPPNLISTPADSADAILSRFSDAGFAAVEVV

GluMnP (131) FGDYIQFAGAVGAANCNGGPQLSFFAGRSNDSQPSPPNLISTPADSADAILSRFSDAGFAAVEVV

GfoMnP (131) FGDFIQFAGAVGVANCNGGPQLSFFAGRSNDSQPSPPNLVPLPSDSADSILSRFSDAGFASVEVV

GapMnP (131) FGDFIQFAGAVGVANCNGGPQLSFFAGRSNDSQPSPPNLVPLPSDTADTILSRFSDAGFDAVEVV

GauMnP (131) FGDFIQFAGAVGVANCNGGPQLSFFAGRSNDSQPSPPNLVPLPSDTADTILSRFSDAGFDSVEVV

**B A**

*** ** * ** * * ***

GluMnP1(196) WLLVSHTVGAQHAVVPSIPGSPFDSTPSDFDAQFFVETMLNGTLVPGNGLQNGEVNSPYPGEFRL

GluMnP (196) WLLVSHTVGAQHAVDPSIPGSPFDSTPSDFDAQFFVETMLNGTLVPGNGLQNGEVNSPYPGEFRL

GfoMnP (196) WLLVSHTVGSQNTVDPSIPGAPFDSTPSDFDAQFFVETMLNGTLVPGDALHDGEVNSPYPGEFRL

GapMnP (196) WLLVSHTVGSQNTVDSSIPGAPFDSTPSDFDAQFFVETMLNGTLVPGNGLQDGEVLSPYPGEFRL

GauMnP (196) WLLVPHTVGSQNTVDPSIPGAPFDSTPSDFDAQFFVETMLNGTLVPGNGLQDGEVLSPYPGEFRL

**A HC M HC C C C**

**** * ***

GluMnP1(261) QSDFAHSRDSRTACEWQHMIADRANMLEKFQVTMLKMSLLGFDQTTLTYCSDVIPAATGTVKDPF

GluMnP (261) QSDFALSRDSRTACEWQHMIADRANMLEKFQVTMLKMSLLGFDQTTLTDCSDVIPAATGTVKDPF

GfoMnP (261) QSDFALSRDSRTACEWQKMIADRANMLEKFEVTMLKMSLLGFNQSMLTDCSDVIPTATGTVQDPF

GapMnP (261) QSDFALSRDSRTTCEWQKMIADRANMLEKFEITMLKMSLLGFDQSALTDCSDVIPTATGTVQDPF

GauMnP (261) QSDFALSRDSRTTCEWQKMIADRANMLEKFEITMLKMSLLGFDQSALTDCSDVIPTATGTVQDPF

**HA B B**

*****

GluMnP1(326) LPAGLTTDDLQPACSSTRFPTVSAVAGAVTSIAAVPLNS

GluMnP (326) LPAGLTTDDLQPACSSTPFPTVSAVAGAVTSIAAVPLNS

GfoMnP (326) IPAGLTVDDLQPACSSTAFPTVNTVAGAVTSIPAVPLNS

GapMnP (326) IPAGLTVDDLQPACSSSAFPTVTTVAGAVTSIPAVPLNS

GauMnP (326) IPAGLTVDDLQPACSSSAFPTVTTVAGAVTSIPAVPLNS

**B**
